# Inactivated herpes simplex virus-1 vaccine formulated in aqueous and alcoholic extracts of propolis boosts cellular and IgG responses

**DOI:** 10.22038/IJBMS.2024.75158.16289

**Published:** 2024

**Authors:** Sanaz Mojarab, Pegah Karimi, Delavar Shahbazzadeh, Majid Moghbeli, Kamran Pooshang Bagheri, Arezoo Beig Parikhani, Rada Dehghan, Ehsan Zafari, Amir Moravej, Mohammad Hassan Pouriayevali, Seyedeh Franak Mirtalebi, Mahdi Pakjoo, Shaghayegh Yazdani, Meghdad Abdollahpour-Alitappeh, Mehdi Mahdavi

**Affiliations:** 1 Advanced Therapy Medicinal Product (ATMP) Department, Breast Cancer Research Center, Motamed Cancer Institute, Academic Center for Education, Culture, and Research (ACECR), Tehran, Iran; 2 Department of Biology, Islamic Azad University, Damghan Branch, Damghan, Iran; 3 Department of Biochemistry, Faculty of Basic Sciences, Islamic Azad University, Central Tehran Branch, Tehran, Iran; 4 Venom and Biotherapeutics Molecules Laboratory, Medical Biotechnology Department, Biotechnology Research Center, Pasteur Institute of Iran, Tehran 1316943551, Iran; 5 Department of Microbiology, Razi Vaccine and Serum Research Institute, Agricultural Research, Education and Extension Organization (AREEO), Karaj, Iran; 6 COVID-19 National Reference Laboratory, Pasteur Institute of Iran, Tehran, Iran; 7 Department of Arboviruses and Viral Hemorrhagic Fevers (National Reference Laboratory), Pasteur Institute of Iran, Tehran, Iran; 8 Department of Microbiology, Zanjan Branch, Islamic Azad University, Zanjan, Iran; 9 Department of Microbiology, Faculty of Advanced Science and Technology, Tehran Medical Sciences, Islamic Azad University, Tehran, Iran; 10 Department of Microbiology, Faculty of Medicine, Tehran Medical Sciences, Islamic Azad University, Tehran, Iran; 11 Cellular and Molecular Biology Research Center, Larestan University of Medical Sciences, Larestan, Iran; 12 Recombinant Vaccine Research Center, Tehran University of Medical Sciences, Tehran, Iran; 13 Immunotherapy Group, The Institute of Pharmaceutical Science (TIPS), Tehran University of Medical Science, Tehran, Iran; # These authors contributed eqully to this work

**Keywords:** Adjuvant, HSV-1, Propolis, Th1, Vaccine potency

## Abstract

**Objective(s):**

In this study, the adjuvant activity of aqueous and alcoholic extracts of propolis was examined on the inactivated herpes simplex virus-1 (HSV-1*)*.

**Materials and Methods:**

BALB/C mice were administered with inactivated (HSV-1; the KOS strain) plus alcoholic and aqueous extracts, followed by assessment of the cellular and humoral immune responses.

**Results:**

Alcoholic and aqueous extracts, as an adjuvant, revealed a significant increase in lymphocyte proliferation and cytotoxic T lymphocyte (CTL) responses versus the HSV-1 group. In addition, HSV-1 plus alcoholic extract showed a remarkable increase in IFN-γ cytokine and IFN-γ/IL-4 ratio. On the other hand, both alcoholic and aqueous extracts in the HSV-1 vaccine suppressed the IL-4 cytokine response as compared with the HSV-1 vaccine. In addition, HSV-1 plus alcoholic extract showed a significant increment in IgG_1_, IgG_2a_, and IgG_2b_ isotypes as compared with the HSV-1 vaccine.

**Conclusion:**

Propolis extracts seem to modulate the immune response against inactivated HSV-1 model and can be used as a suitable vaccine adjuvant or a component of a complex adjuvant against infectious diseases.

## Introduction

Although vaccines can decrease the incidence of infectious diseases, some vaccines lack sufficient efficacy, highlighting the need for improving the efficacy of such vaccines. Adjuvants are substances combined with candidate antigens to improve the vaccine efficiency. There are natural compounds with immunomodulation properties such as propolis (1, 2). Propolis is a resin-brown substance that is collected by worker bees (*Apis mellifera meda*) and was shown to have boosting effects on Th1 and humoral responses in several vaccine models (3-6). In the present study, inactivated HSV-1 was formulated in aqueous and alcoholic extracts of propolis derived from the Iranian Honey bee, as an adjuvant, and then the immune responses of the newly formulated vaccine were assessed in mice.

## Materials and Methods

Propolis, kindly gifted by Dr. Pouria Ghasemi from the Pasteur Institute of Iran, was homogenized either in distilled water or ethanol (95-96%) for about 2 weeks (7). The vaccine contained 5 μg of inactivated HSV-1 in 200 μl of phosphate-buffered saline (PBS) buffer with or without 5 mg of the aqueous extract of propolis. Six- to 8-week-old BALB/C mice were divided into six groups containing 6 mice in each one. Groups 1 through 6 received 5 μg inactivated HSV-1+5 mg of alcoholic extract, 5 μg inactivated HSV-1+5 mg of aqueous extract, 5 μg inactivated HSV-1, 5 mg of alcoholic extract, 5 mg of aqueous extract, and PBS as control groups, respectively. 

Experimental mice were immunized subcutaneously on days 0, 14, and 28. Two weeks after the third immunization, 3×10^5^ of spleen cells were dispensed into 96-well flat-bottom culture plates and stimulated with 1 μg/well of inactivated HSV-1 virus for 72 hr. Afterward, blood samples were collected from the mice 2 weeks after the third injection through the retro-orbital puncturing of mice for antibody assay. The lymphocyte proliferation was then assessed using a BrdU kit (Roche, Germany). Interferon-gamma (IFN-γ) and interleukin 4 (IL-4) cytokines were measured on the spleen cell culture supernatant using commercial Enzyme-linked immunosorbent assay (ELISA) kits (Quantikine, R&D Systems, USA) according to the manufacturer’s instructions. After 36 hr of antigen stimulation, the culture supernatant was collected and used for Granzyme B (Gr-B) assay using an ELISA kit (eBiosystem, USA). In order to evaluate the immunoglobulin G (IgG) response, HSV-1 inactivated viruses were coated on 96-well ELISA plates (Greiner, Germany) and incubated for 24 hr at 4 ^°^C. The wells were washed with PBS containing 0.05% Tween 20 (washing buffer) and blocked with 5% skimmed milk in washing buffer for 60 min at 37 ^°^C (blocking buffer). Subsequently, 100 μl of 1/50 to 1/6400 dilutions of sera were added into each well and incubated for 90 min at 37 ^°^C. The wells were washed before incubation for 90 min with 100 μl of 1/10000 dilution HRP-conjugated anti-mouse antibody (Sigma, USA). Following five-time washing, the wells were incubated in the dark for 30 min with 100 μl of the TMB substrate, the reaction was stopped with 2N H_2_SO_4_, and the color density was read with an ELISA plate reader at 450 nm (BioTek, USA). Meanwhile, specific IgG_1_, IgG_2a_, IgG_2b_, and IgM subclasses were evaluated using an ISO-2 kit according to our optimized ELISA (Sigma, USA)(8). Lymphocyte proliferation assay, cytokines, and Gr-B assay were performed in duplicate, and antibody ELISA was performed in triplicate. The Mann-Whitney U test was used in all statistical analyses. *P-*values less than 0.05 were considered statistically significant in all circumstances.

## Results

**Figure 1:**
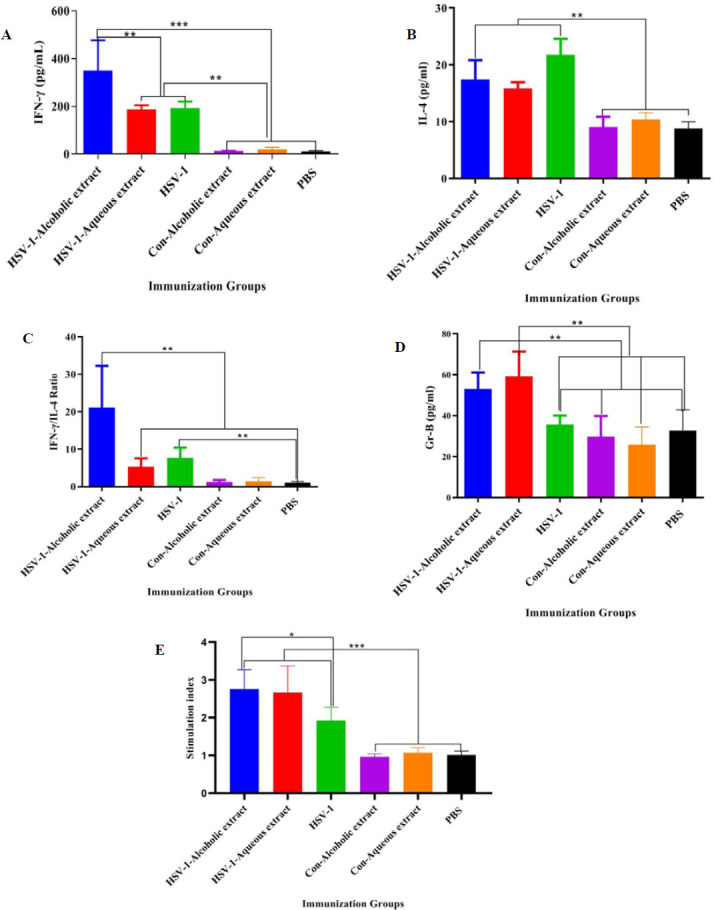
Results from lymphocyte proliferation of experimental mice against herpes simplex virus-1 (HSV-1) vaccine

Mice immunized with the HSV-1 vaccine alone or with propolis alcoholic and aqueous extracts showed a significant increase in lymphocyte proliferation as compared with the control groups. At the same time, HSV-1 with propolis alcoholic extract showed a significant increment as compared with the HSV-1 group (*P*=0.0152)(Figure 1).

**Figure 2:**
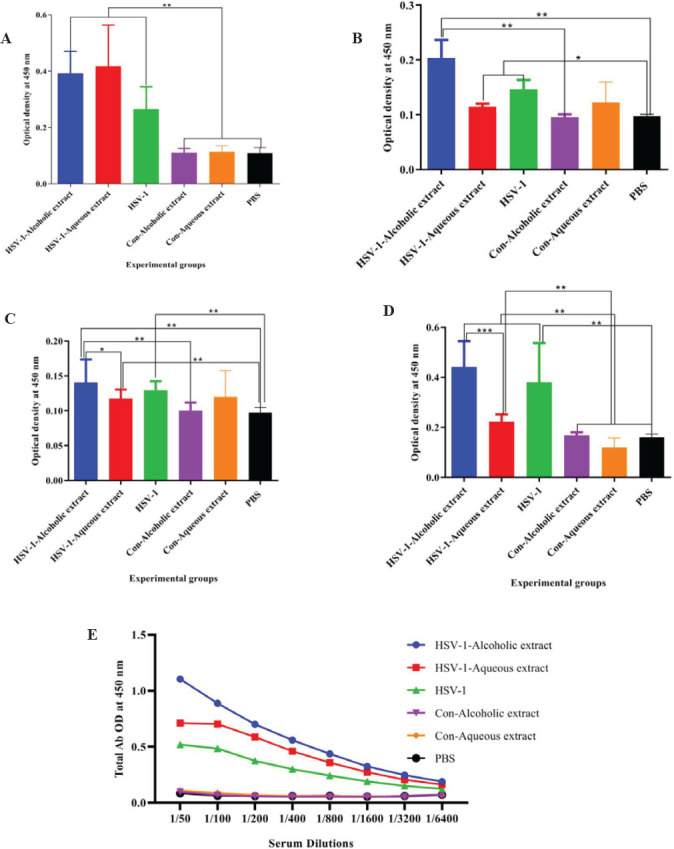
Results from (A) IFN-γ, (B) IL-4 cytokines, and (C) IFN-γ /IL-4 ratio in the experimental groups

**Figure 3:**
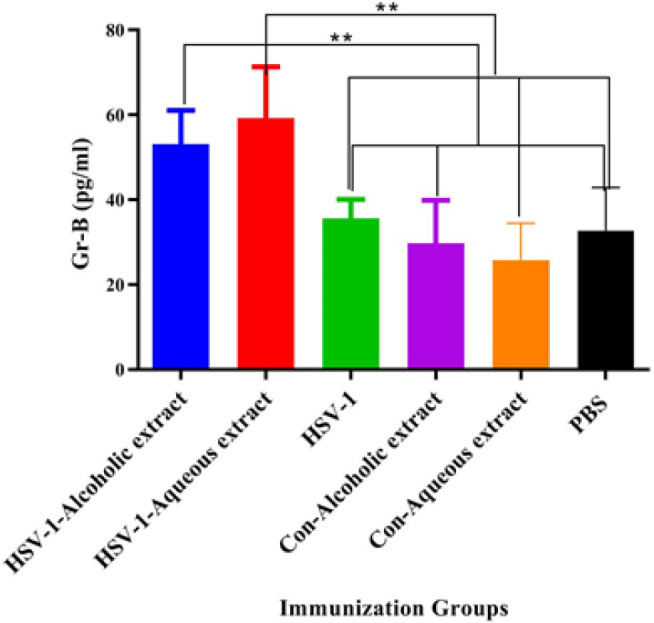
Results from cytotoxic T lymphocyte (CTL) activity in the experimental groups

IFN-γ cytokine response in all vaccine groups showed a significant rise as compared with the control groups (*P*<0.0002). Additionally, the vaccine with alcoholic extract showed a remarkable increment as compared with the vaccine plus aqueous extract and HSV-1 vaccine (*P*<0.0010) (Figure 2A). Results from the IL-4 cytokine response in HSV-1 plus alcoholic and /or aqueous extracts as well as the HSV-1 vaccine showed a significant rise as compared with the control groups (Figure 2B). Furthermore, the IFN-γ/IL-4 ratio in HSV-1 plus alcoholic extract showed a remarkable increase as compared with all other experimental groups (Figure 2C). Results from Gr-B release as a criterion of cytotoxic T lymphocytes (CTL) activity in the vaccine with alcoholic extract with or without aqueous extracts led to a significant rise as compared with the control groups and the HSV-1 vaccine group (Figure 3).

**Figure 4:**
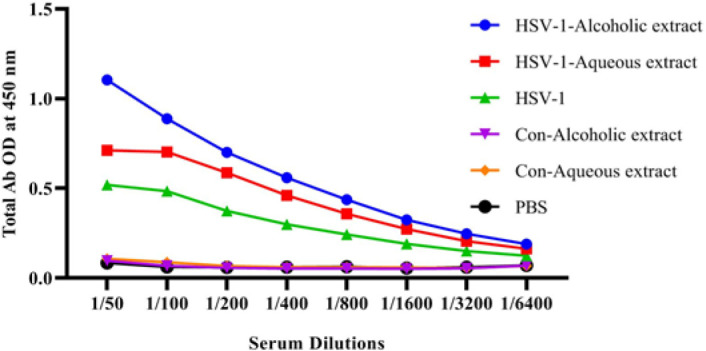
Results from specific total IgG antibody

Results from specific total IgG antibody showed a significant total IgG increment of mice immunized with the HSV-1 vaccine plus alcoholic extract as compared with the alcoholic extract control and PBS group at the dilutions of 1/50 to 1/1600 (Figure 4).

**Figure 5:**
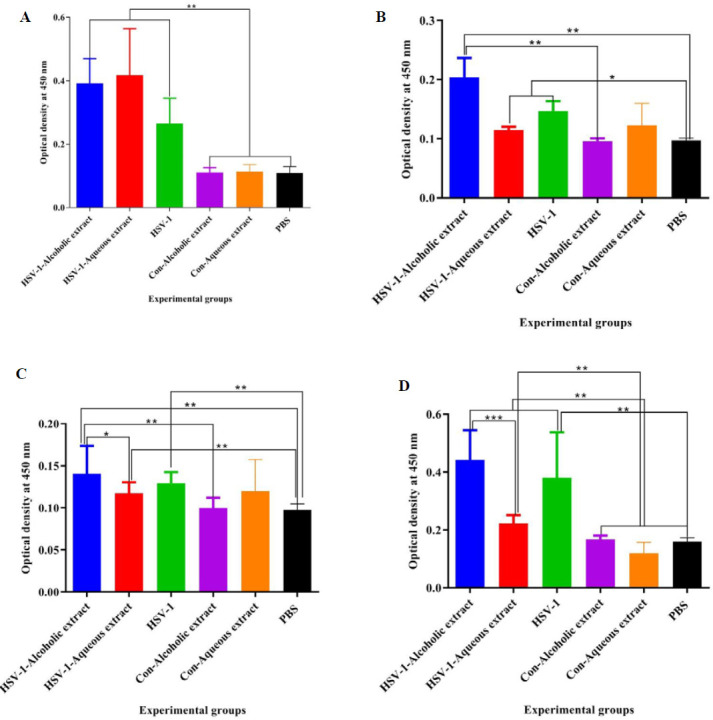
Results from specific (A) IgG_1_, (B) IgG_2a_, (C) IgG_2b_, and (D) IgM isotype antibodies in the experimental mice

Results from specific IgG_1_ isotype in the vaccine with alcoholic extract with or without aqueous extracts and HSV-1 vaccine revealed a remarkable rise as compared with the control groups (Figure 5A). The IgG_2a_ isotype in the vaccine with alcoholic extracts showed a significant increase as compared with the alcoholic extract control and PBS group, while HSV-1 plus aqueous extract and HSV-1 vaccine revealed a remarkable increase versus PBS (Figure 5B). 

Results from specific IgG_2b_ isotype in the vaccine with alcoholic extracts showed an increase as compared with the HSV-1 plus aqueous, alcoholic extract control, and PBS group. Mice immunized with the HSV-1 vaccine plus alcoholic extract showed a significant increment in the IgG_2b_ response versus the HSV-1 plus aqueous extract group (*P*=0.0371)(Figure 5C). 

Results from the specific IgM isotype in the vaccine with alcoholic extract with or without aqueous extracts and HSV-1 vaccine revealed an outstanding increase as compared with the control groups. Additionally, mice immunized with *the *HSV-1 vaccine plus alcoholic extract showed a significant increment in the IgM response versus the HSV-1 plus aqueous extract group (Figure 5D).

Alcoholic and aqueous extracts of propolis in the vaccine formulation showed a significant increase against the HSV-1 vaccine (*P<*0.028)

(A) Immunization of herpes simplex virus-1 (HSV-1) vaccine plus alcoholic extract showed a significant rise in IFN-γ cytokine versus HSV plus aqueous extract and *HSV-1* vaccine (*P=*0.014 and *P=*0.016, respectively). (B) Immunization with *HSV-1* vaccine formulated in alcoholic and/or aqueous extracts suppressed IL-4 cytokine response versus the HSV-1 vaccine (*P=*0.01 and *P=*0.063, respectively). (C) Results from the IFN-γ/IL-4 ratio in the HSV-1 plus alcoholic extract group showed a remarkable increase versus all other experimental groups (*P<*0.0221)

Immunization with the vaccine plus alcoholic and vaccine plus aqueous extracts showed a significant increase compared with the *Herpes Simplex Virus-1* (*HSV-1)* vaccine group (*P=*0.0005 and *P=*0.0006, respectively)

Immunization with the *Herpes Simplex Virus-1* (*HSV-1)* vaccine plus alcoholic extract showed a significant increase versus the *HSV-1* vaccine at dilutions of 1/50 to 1/800 (*P=*0.0255). In addition, immunization with HSV-1 plus aqueous extract at dilutions of 1/50 to 1/200 showed a significant increment versus the *HSV-1* vaccine group (*P<*0.0100). Furthermore, the *HSV-1* vaccine plus alcoholic extract revealed a remarkable increase versus the *HSV-1* plus aqueous extract at dilutions of 1/50 and 1/100 (*P<*0.0384)

(A) Immunization with the herpes simplex virus-1 (HSV-1) vaccine in alcoholic and/or aqueous extracts formulation showed a significant increase in IgG_1_ isotype versus the *HSV-1* vaccine group (*P=*0.005 and *P=*0.0078, respectively). (B) Results from the specific IgG_2a_ isotype revealed that immunization with *HSV-1* plus alcoholic extract showed a significant increase versus HSV-1 plus aqueous extract and *HSV-1* vaccine group (*P=*0.0001 and *P=*0.0002, respectively).(C) Results from the specific IgG_2b_ isotype revealed that immunization with HSV-1 plus alcoholic extract showed a significant rise versus the HSV-1 plus aqueous extract group (*P=*0.0371). (D) Immunization with HSV-1 vaccine plus alcoholic extract showed a remarkable increase in IgM response versus the *HSV-1* plus aqueous extract group (*P=*0.0001)

## Discussion

Studies showed that propolis could modulate immune responses in several aspects, such as Th1 and Th17 polarization, CTL improvement, and induction of IgG response (5, 9, 10). In this study, lymphocyte proliferation as a criterion of cellular immune response showed that both alcoholic and aqueous extracts of propolis improved T-cell responses. In a study carried out by Conti *et al*., propolis could stimulate lymphocyte proliferation responses through the modulation of dendritic cells (10). Of note, some bodies of evidence showed the potency of propolis extracts in triggering T cell proliferation (7, 11, 12), which is in parallel to our findings, demonstrating the ability of both alcoholic and aqueous extracts of propolis to stimulate cellular immune responses. It is clear that cellular immunity is critical in controlling viral infections (13) and this is an advantage for propolis in the vaccine formulation. 

Cytokine IL-4, as a Th2 (T helper type 2), and IFN-γ, as a Th-1 response (14), indicated that alcoholic extract in the HSV-1 vaccine formulation improved the IFN-γ cytokine response. Meanwhile, both alcoholic and aqueous extracts in the HSV-1 vaccine suppressed IL-4 cytokine release. These findings showed the potency of alcoholic and aqueous extracts in the induction of the Th1 (T helper type 1) response either by increasing IFN-γ cytokine responses or through suppression of IL-4 cytokine responses. However, the alcoholic extract showed a more robust Th1 response.

In a study, the ethanolic extract of propolis and Alum, as an adjuvant, plus the inactivated Suid herpesvirus type 1 (*SuHV-1*) vaccine showed a Th1 response (15). In a study performed by Bezerra *et al*., a recombinant protein rCP01850 plus Brazilian red propolis showed a Th1 response and protection against *Corynebacterium pseudotuberculosis* challenge in mice (16). All of these studies confirmed the immunomodulatory effect of propolis extracts in the induction of the Th1 immune response as demonstrated in this study. Additionally, the IFN-γ/IL-4 ratio, as another parameter of confirming Th1 response, showed the highest level in the HSV-1 vaccine plus the alcoholic extract group which showed a Th1 response.

Results from CTL activity showed a significant rise in the HSV-1 vaccine plus alcoholic and aqueous extracts versus the HSV-1 vaccine. A study conducted by Onur *et al*. on breast cancer-bearing mice showed that propolis induced CTL activity of mice as well as improving the anti-tumor CTL response when used in combination with acidophilus milk, suggesting that propolis extracts are useful as an adjuvant in the vaccine formulation to induce cellular immune for cancer immunotherapy (17). It is well-known that Th1 and CTL responses are important in controlling tumor growth (18). The specific IgG response in the HSV-1 vaccine plus both alcoholic and aqueous extracts was able to boost humoral immune responses. Several studies confirmed the boosting activity of propolis extracts on the antibody response in vaccine models (16, 19). Improvement of humoral immune responses by propolis extracts is an important property, because of the important role of humoral immune response in the neutralization of viruses and clearance of the body (20).

Results from antibody isotypes showed that alcoholic extract of propolis increased IgG_1_, IgG_2a_, and IgG_2b_ while not showing a positive effect on IgM response isotype responses in comparison with the HSV-1 group. Nevertheless, the aqueous extract revealed a boosting effect only on the IgG_1_ response in comparison with the HSV-1 group. This finding showed that the bioactivity of these extracts on the humoral immune response is different and alcoholic extract is more efficient than aqueous extract in the induction of multi-isotype humoral immune responses. Induction of multi-isotype humoral immune response is very important because each specific isotype of antibody has a special function in the humoral immune response, and stimulation of antibody response with different isotypes means higher bioactivity of humoral immune responses.

## Conclusion

Alcoholic and aqueous extracts of propolis boosted cellular and humoral immune response versus HSV-1 vaccine which may candidate these components as valuable vaccine adjuvants.
